# Revisiting 3Rs: rethinking replacement and new approach methodologies

**DOI:** 10.3389/ftox.2025.1664209

**Published:** 2025-11-14

**Authors:** Jessica Rosolowski, Tilo Weber, Atena Malakpour-Permlid, Stina Oredsson

**Affiliations:** 1 Animal Welfare Academy of the German Animal Welfare Federation, Neubiberg, Germany; 2 Department of Health Technology, Center for Intelligent Drug Delivery and Sensing Using Microcontainers and Nanomechanics, Technical University of Denmark, Kongens Lyngby, Denmark; 3 Department of Biology, Lund University, Lund, Sweden

**Keywords:** replacement, new approach methodologies, cruelty-free, xeno-free, key actions

## Introduction

The 3Rs concept – replacement, reduction, and refinement – a framework for animal research introduced in 1959 by Russel and Burch, is widely adopted amongst researchers, authorities, and industries. The implementation of reduction and refinement has decreased the suffering of animals for scientific purposes throughout the world by promoting frameworks for reducing the number of animals used in experiments and the severity of their individual suffering. These are important ethical principles that accompany the transition to a full replacement of practices that inflict pain, suffering, and harm on animals in science. In the following, we discuss how different terms are used in the context of replacement and how their definition may impact progress toward the complete replacement of animal use.

## Terminology of an ambitious paradigm change

“Replacement” is a term that may seem straightforward – using methods other than animal-based ones to achieve the same scientific aim. Among these replacement strategies, cell-based methods are widely used to study human biology. Despite current limitations of cell-based assays in replicating the overall complexity of the human body (Lutolf et al., 2024), there is growing demand and scientific evidence for replacement approaches as means of reaching the goal of human-relevant model systems. At the same time, the concept of replacement requires further reflection in light of the more recently established terminology, “new approach methodologies,” abbreviated as NAMs, or “non-animal methods,” which is referred to by the same acronym (Nelson et al., 2024). These terms refer to methods that are free from animal experimentation. However, these terms might also give the impression that animals are not being used at all, which may not be accurate since animal-derived materials are still part of many methods aimed at replacing animal experiments (Cassotta et al., 2022; Duarte et al., 2023). We seek to raise awareness of this misconception, which fortunately also presents a valuable opportunity for advancing ethical considerations concerning animal use in science. We encourage the scientific community to head toward totally animal-free practices – replacing not just experimentation on live animals but also the reliance on materials sourced from live or dead animals.

The transition to NAMs presents the only feasible strategy for replacing procedures on live animals while also promoting innovation in science. Statistical data indicating a clear global shift are not available yet, although across the European Union (EU), the use of animals for scientific purposes has declined by 11% over the past 20 years, while the development and use of NAMs have been increasing exponentially, especially since the mid-2010s (Taylor, 2024). Publication numbers for countries with the highest numbers of animal use (China, France, Germany, United Kingdom, USA) further indicate that, proportionally, the reliance on animals in science is decreasing (Taylor et al., 2024).

Since this transition is still taking shape, it is a suitable time to define and agree on the end goal of full replacement, including the elimination of animal-derived materials. It may be an ideal that is not yet feasible in all areas, but wherever possible, full replacement should already be implemented. There is no doubt that significant efforts are being made through various research funding bodies and industry initiatives to develop NAMs that avoid the use of animals (NC3Rs, 2024; European Commission, 2025). Our hope is that these efforts will also strive toward a full replacement of animal-derived materials. To track such progress, quantitative data on both the use of animals in the production of laboratory materials as well as for the development of the animal-free NAMs market should be documented and published.

## Implications for product labeling

The implications of using animal-derived laboratory materials are enormous when considering just how many materials and products must be tested for safety and efficacy before being launched on the market. From pharmaceuticals to novel foods, cleaning reagents, and pesticides – almost all chemicals that come into contact with humans or the environment have had to undergo such testing.

One particularly prominent example is the cosmetics market. The EU implemented a ban on animal testing for cosmetic products and cosmetic ingredients 15 years ago and prohibited the sale of cosmetics that had been tested on animals outside of the EU via its Regulation (EC) No 1223/2009 of the European Parliament. Despite shortcomings in their implementation (Knight et al., 2021), these bans have promoted the development of a significant number of OECD-accepted test guidelines for the evaluation of various toxicities without using animals. While this is a major achievement, it is a well-known fact that animal-derived materials are still being used in most of these tests. Moreover, many cosmetic products are subsequently labeled as “cruelty-free” – a term that was established to imply that no live animals were used in the testing process. This term in itself is vague and may be confusing for various reasons since cruelty is defined rather subjectively than scientifically and differs depending on the specific context, i.e., the mere sourcing of some raw materials for commercial products might harm local communities and ecosystems and can therefore be considered cruel (ten Kate et al., 2016; Giri and Singh, 2023).

Another issue is that the sourcing of animal-derived materials which are used in NAMs is not always conducted under ethical conditions and could therefore be considered cruel, as is the case with fetal bovine serum (FBS), the most common animal-derived laboratory material, followed by antibodies, dissociation enzymes, growth factors, and coating materials (Cassotta et al., 2022). FBS is obtained through procedures that involve the suffering of pregnant cows and their fetuses (Weber et al., 2021). The use of animals as “production plants” for antibodies and basement membrane extracts also raises serious animal welfare concerns (Hendriksen, 1998; Berg and Kurreck, 2021). While certain materials are obtained as by-products from slaughtered animals, they nonetheless remain ethically contentious due to their sourcing from industrial animal agriculture. The term “cruelty-free” is also increasingly employed by initiatives aimed at reforming farming systems, thereby broadening its ethical scope beyond the context of animal testing (Humane Foundation, 2025). In addition, cosmetic products may be labeled “cruelty-free” despite containing animal-derived ingredients as part of their formulation and thus not being vegan.

Therefore, the label “cruelty-free” can be misleading, as it does not always reflect ethical manufacture and testing of a product. It is important that these issues are communicated to scientists, industries, politicians, authorities, regulators, and, in particular, to the general public. We need to reflect on the way we use the terms “replace,” “NAMs,” and “cruelty-free” to eliminate any room for (mis-) interpretation (Sheehan and Lee, 2014). An essential requirement for using such terms and labels must be full transparency regarding their definition and scope. Consumers who purchase products such as cosmetics may remain unaware of hidden animal welfare implications – even for products which are labeled both “cruelty-free” and “vegan”. If provided with clear information, the general public might add to the pressure on industries and politicians to implement consistent animal protection practices – thus becoming an active voice for change.

## Re-defining language to advance the 3Rs

The terminology around the use and replacement of animals matters for ensuring clear scientific communication, providing transparent information to the general public, and, most importantly, establishing consistency in regulatory and legal contexts. Table 1 provides an overview of how relevant terms can be distinguished, while Table 2 offers recommendations for their potential re-definition or clarification.

**TABLE 1:** Distinction of commonly used terms based on different criteria of animal use.

Term	May contain animal-derived product ingredients	May involve animal testing	May involve animal-derived laboratory materials
Cruelty-free	Yes	No	Yes
NAMs^a^	Not applicable to products	No	Yes
Vegan	No	Yes	Yes
Xeno-free	Not applicable to products	No (in human-relevant science)	No (in human-relevant science)

^a^ NAMs, New Approach Methodologies.

**TABLE 2:** Current uses and recommendations for key terms in the context of the 3Rs.

Term	Current use	Recommendation
Cruelty-free	Used in product labelling and marketing to indicate that a product has not been tested on animals	Due to the broad scope and subjective nature of the term “cruelty”, it should be replaced with more specific phrases, i.e., “not tested on animals” or “animal testing free”. The use of live animals must be avoided for the testing of both ingredients and final products. To achieve consistent ethics, such products should ideally be vegan and tested in a xeno-free manner
NAMs	Describes methodologies designed to replace, reduce, or refine the use of live animals. Exact definitions vary and may refer to “new approach methodologies,” “non-animal methods,” or a combination of both as “non-animal new approach methodologies” (Courtot et al., 2025)	A harmonized definition should be established, referring to methods or scientific approaches which avoid the use of live animals, and ideally also avoid the use of animal-derived materials (in the context of human-relevant science), using fully defined and reproducible systems
Vegan	Used in product labelling and marketing to indicate that a product does not contain animal-derived ingredients	The definition should be communicated more clearly in terms of animal testing being out of its scope (unless stated otherwise in the conditions of specific labels). To achieve consistent ethics, vegan products should ideally avoid animal testing for ingredients or end products as well as being tested in a xeno-free manner
Xeno-free	Proposed to describe fully animal-free technologies which avoid the use of both live animals and animal-derived materials (Ali et al., 2025)	The term is useful for referring to species-specific approaches, but requires specification of its context, i.e., xeno-free methods and materials for human-relevant research and testing avoid both the use of live animals and animal-derived materials

For the scope of this discussion, we focus our recommendations on human-related science and cell lines, while acknowledging that research and testing in areas such as environmental and veterinary sciences may involve deviating requirements as well as increasing relevance in line with the One-Health approach. In this context, we define the goal as species-specific test systems (see xeno-free, Table 2) which may only utilize animal-derived materials which do not cause further pain, suffering, or harm to animals (i.e., long-established immortalized animal cell lines or recombinant animal antibodies).

## The case of the fetal bovine serum black box

FBS serves as a specific example for the problematic use of animal-derived materials in cell culture, as well as for the efforts made to leave it behind (Weber et al., 2025). FBS supplementation of growth medium is associated with issues in terms of safety, reproducibility, and ethics, the most significant ethical concern being the sourcing of FBS from calf fetuses. Safety and reproducibility problems with FBS concern the large variation between batches, risk of being contaminated by bacteria, viruses, prions, and environmental toxins, its unknown and inconsistent composition, as well as the fact that it is not human-based despite being used in human-relevant research.

The unknown composition of FBS means that cells are cultured in a “black box.” There is ongoing research to formulate novel cell culture media that do not contain FBS or other animal-derived components to overcome these problems. Unfortunately, the “black box” problem persists as many of these alternative media are proprietary, meaning their compositions are not disclosed. To address this, chemically defined cell culture media with fully disclosed formulations are being developed and published (Rafnsdóttir et al., 2023; Weber et al., 2024; Mogilever et al., 2025; Nessar et al., 2025; Oredsson et al., 2025). Likewise, we encourage all commercial producers of cell culture media to disclose the full contents and compositions of their products. Cells are inherently complex and can be described as their own biological “black boxes” due to the vast unknowns in their internal mechanisms. While we may not yet fully illuminate the cells themselves, we can at least shed light on their immediate surroundings. Illuminating the culture media will support scientists in uncovering the inner workings of the cells (Weber et al., 2025). Just like animal-derived materials can alter cell behavior, their replacement may similarly affect cellular responses. Hence, validation studies are required, particularly for sensitive applications, to determine whether a specific animal-free material can adequately support the biological functions of the cells that are being investigated. For this purpose, data and insights obtained from human cells *in vivo* can serve as a (gold) standard for human cell tests *in vitro*. Transitional phases will be required, but ultimately, a more transparent and comprehensible scientific process can be achieved.

Finally, we do not believe that disclosing the composition of a cell culture medium will result in a decrease in its sales. In fact, the demand might increase since scientists may find greater confidence in using a product of known composition. The adaptation of disclosed cell culture media without animal-derived materials will reallocate the billions of dollars spent yearly on purchasing products such as FBS, which creates great opportunities for companies to focus on the production and sale of animal-component-free media.

## Key actions to advance scientific and ethical progress

The implementation of practices and principles that fully align with the ideal of research and science being entirely free from animal suffering is an ambitious undertaking. Changing the *status quo* presents significant challenges for industry, academia, and policymakers, requiring both time and the allocation of adequate resources. Establishing a true gold standard for validating animal-free methods and xeno-free cell media can only be achieved through data and insights derived from human-relevant systems. Meaningful progress depends on developing tools that enable us to track advances in replacing animal use in science. We acknowledge these challenges but are confident that growing awareness and a shared vision of our goals provide a strong foundation for future progress.

In conclusion, we propose the following systemic actions toward a more ethical science:

• Standardization, harmonization, and transparency: Definitions of key terminology should be established and implemented on a global scale. These should be communicated to the public to educate on the ethical impact of animal-derived materials, thus allowing for more conscious consumer choices in the future.
• Re-evaluation of regulatory frameworks: Current OECD Guidelines for the Testing of Chemicals should be updated to prioritize animal-component-free, disclosed media in regulatory testing. Specific (financial) support for this transition should be provided. Collaboration between academia and industry should be encouraged to facilitate the development and validation of animal-free methods.
• Encouragement of animal-free science: Research focused on replacement and NAMs should avoid animal-derived materials. Respective consideration should be applied when allocating project funding. The Directive 2010/63/EU should be updated to consider animal-derived materials. The introduction of similar policies on a global scale would increase accountability and help foster a long-term commitment to adopt animal-free practices. A centralized database on animal-derived materials and their animal-free alternatives should be established to communicate new opportunities for replacement more efficiently and to document progress being made.
